# Unraveling the hidden link between asthma and depression among University students in Bangladesh

**DOI:** 10.1371/journal.pone.0325348

**Published:** 2025-05-30

**Authors:** Tafajjal Islam, Masom Mia, Md. Abdul Hannan Mondal, Mohammad Jahid Hasan, M. Tasdik Hasan, Md. Golam Hossain

**Affiliations:** 1 Department of Psychology, Faculty of Biological Science, University of Rajshahi, Rajshahi, Bangladesh; 2 Health Research Group, Department of Statistics, University of Rajshahi, Rajshahi, Bangladesh; 3 Department of Clinical Psychology, Faculty of Biological Science, University of Rajshahi, Rajshahi, Bangladesh; 4 Pi Research Consultancy Center, Dhaka, Bangladesh; 5 Action Lab, Faculty of Information Technology, Monash University, Melbourne, Australia; University of Texas at Brownsville: The University of Texas Rio Grande Valley, UNITED STATES OF AMERICA

## Abstract

**Background:**

Asthma is a severe long-term respiratory and multidimensional disease. It affects a large proportion of people worldwide. Knowledge regarding asthma and its impact on depression among university students in Bangladesh is very limited. The objective of this study was to investigate asthma, as well as to explore the relationship between asthma and depressive symptoms among university students in Bangladesh.

**Methods:**

This study employed a cross-sectional design. Data was collected from Rajshahi University, Bangladesh. A total of 400 university students were recruited for this study. A semi-structured questionnaire was utilized to gather information from the participants. The primary outcome variable was asthma, which was assessed with the question: “Are you suffering from asthma (diagnosed by a doctor within the past year)?” Depressive symptoms were measured using the nine-item Patient Health Questionnaire (PHQ-9). Factors influencing asthma and its association were examined using a binary logistic regression model.

**Results:**

The study revealed that 53 (13.3%, 95% CI: 10.1% − 17.0%) of the students were suffering from asthma. Three significant predictors of asthma among university students were found: (i) having a family history of asthma (p < 0.01), (ii) being underweight (p < 0.01), and (iii) being born as a second or later child (p < 0.01). Findings also revealed that 48.8% of students exhibited symptoms of depression, with a significantly higher prevalence among female students compared to males (65.3% vs. 38.8%). Having asthma was found to be significantly associated with increased odds of depression (OR = 2.65, p < 0.002). Students with a history of asthma had 165% of more likelihood to depressive symptoms compared to their non-asthmatic peers suggesting a significant association between depressive symptoms and asthma in this population.

**Conclusion:**

A considerable number of university students in Bangladesh suffer from asthma, with several modifiable factors such as depressive symptoms associated with the condition. The findings of this research will assist health professionals, policymakers, and university authorities in addressing the health challenges faced by students.

## Introduction

Asthma is one of the most prevalent respiratory conditions, affecting over 260 million individuals globally and resulting in more than 4.5 million deaths annually, the majority of which are preventable [1]. This number is projected to exceed 400 million by 2025 due to increasing urbanization [2]. Asthma is a complex, multidimensional disorder characterized by interactions among neurological, endocrine, immunological, behavioral, and psychological processes [3]. Over the past 40 years, the global prevalence, morbidity, mortality, and economic burden of asthma have risen significantly [4]. The prevalence of asthma varies by geography and socioeconomic status, with higher incidence rates observed in high-income countries; however, some middle-income and low-income countries also report considerable incidence [5]. Approximately, 90% of individuals with asthma reside in low- and middle-income countries (LMICs) [6].

Bangladesh is a lower middle-income country where asthma is a considerable public health issue, affecting 4.1 million children and 11.6 million adults with limited epidemiological data on its prevalence [7]. The prevalence of asthma in Bangladesh has been estimated in several studies. With a history of 25 years, the prevalence of asthma in both the general and rural populations in Bangladesh has ranged from 4.2% to 9.85%, and the risk factors for asthma outcomes are age, sex, occupation, lifestyle, and socioeconomic and comorbidity factors [7–10]. A previous study of rural communities in Bangladesh revealed the significance of a family history of asthma [10]. A recent review reported a relationship between obesity, smoking status, poor socioeconomic status, sex (females more than men), and the onset of asthma in adults [11]. Study on asthma among university students in Bangladesh is scarce; however, one study involving agricultural university students in Bangladesh was conducted 10 years ago [12]. Some health indicators have improved during the last two decades with increasing education and household wealth indices in Bangladesh [13].

Depressive symptoms are more common in asthma patients than in the general population [14]. Individuals with asthma are more likely to experience depression, similar to other chronic illnesses [15]. Several studies have shown that there is an association between depression and asthma [14,15]. The relationship between depression and asthma is complex, with depression increasing asthma symptoms and vice versa. Depression increases the probability of having asthma by 2-fold [16]. Having both depression and asthma is worse for a person’s health than having depression or asthma alone [17]. Studies have shown that 18.1% of adults with asthma have comorbid depressive disorders [15], and the probability of mental disorders in asthma patients is 1.6 times higher (95% CI, 1.4–1.8) for those with depressive disorders [18]. Many patients report that depression has a negative effect on their asthma control [14]. Previous studies have had limitations, such as small sample sizes and cross-sectional studies, limiting the ability to determine the temporal relationship between depression and asthma [18,19]. The effects of this association are also unclear.

University students with asthma and depression face hardship to realize their potential contributions to the development of their country, particularly in developing nations like Bangladesh, where many sectors require enhancement through the engagement of the youth. It is essential to prioritize the health of university students. Understanding the prevalence of asthma, its associated factors, and its impact on depressive symptoms among university students in Bangladesh is crucial for promoting healthy lifestyles, improving asthma management practices, and reducing the burden of asthma-related morbidity and healthcare costs. This population is particularly vulnerable to lifestyle changes within the academic environment. Therefore, the objective of this study was to assess the prevalence and associated factors of asthma and its relationship with depressive symptoms among university students in Bangladesh.

## Methods

### Study design and settings

This cross-sectional study was conducted among students of Rajshahi University (RU) in Bangladesh from 10 May 2024 to 21 July 2024. The public university is the second-largest non-medical university in Bangladesh, and its students come from different parts of the country. Currently, a total of 37156 students are studying at the university. Of them, approximately 25000 students (18000 males and 7000 females) reside in residential halls inside the campus, and the remaining students reside outside the campus of the university.

### Exclusion criteria

This study exclusively included students who did not have serious illnesses and are currently residing inside the RU campus.

### Sample size determination

The sample size was calculated using the following formula: n = N/(1 + N*d^2^)=394 [20], where n represents the desired sample size, N represents the population size (in this case, 25000), d represents the marginal error (d = 0.05), and the 95% confidence level was considered. The formula estimated that 394 samples were required for the current study; however, we initially considered 420 samples with a 7% absence rate.

### Sampling techniques

Multistage stratified random sampling was used to select samples from the population. There are 17 residential halls (11 for males and 6 for females) in the RU campus. In the first stage, we selected 4 halls for males and 2 halls for females by random sampling using a lottery method. In the second stage, 66 male and 78 female students were selected from each selected male and female hall respectively by random sampling. The total number of samples was 420 (male, 66 × 4 = 264; female, 78 × 2 = 156). All necessary information for sampling was gathered from the administrative offices of the selected halls. Formal approval was taken from the authorities of the selected residential halls for contacting students. Our data collectors arranged a meeting for selected students and discussed the objective of the present study, and obtained written consent from the students who agreed to provide their information; unfortunately, 14 male and 6 female students denied to share their information. Finally, data was obtained from 400 students.

### Data collection procedure

A semi-structured questionnaire was used to collect information from the selected students. The questionnaire was divided into three parts: (i) sociodemographic, lifestyle, and behavioral information; (ii) asthma measurement; and (iii) nine questions from the Patient Health Questionnaire (PHQ-9) to assess depression. First, we prepared a draft English questionnaire and sent it to five experts in public health research to obtain their opinions/suggestions/comments to improve the questionnaire. We followed the experts’ opinions/suggestions/comments and finalized the questionnaire in English. As the mother tongue of the students was Bangla, the English questionnaire was translated to Bangla, and it was checked by a bilingual expert.

### Independent variables

We considered socio-demographics, lifestyle, and behavioral factors as independent variables. For collecting socio-demographic information, our data collectors asked the students to complete a questionnaire regarding their age, sex, academic year, parents’ occupation, parents’ level of education, monthly family income (BDT) and marital status. We measured the students’ height (cm) and weight (kg) by standard instruments. The following behavioral and lifestyle factors were included: fried food intake (frequency per week), red meat intake (frequency per month), physical exercise (hours per week), added salt intake (yes/no), smoking status (yes/no), and substance abuse (yes/no). The independent variables were selected according to the WHO Package of Essential Noncommunicable Disease Interventions (WHO PEN) model [21]. Most of the socio-demographic, lifestyle, and behavioral factors used in previous studies have been reported [8,22].

### Outcome variable

We had two outcome variables in the present study, and the primary outcome variable was asthma, which was measured by the following question: “are you suffering from asthma (diagnosed by a doctor within one year)?” Asthma was identified in subjects who had either an inpatient diagnosis and/or at least one year of diagnosis from outpatient services. On the basis of answers, the students were classified into two classes: not having asthma (No, code = 0) and having asthma (Yes, code = 1) [23]. The secondary outcome variable was depression. According to DSM-IV standards for diagnosis, the Patient Health Questionnaire-9 (PHQ-9) is a common patient self-report symptom assessment survey for depression diagnosis and for measuring depressive symptom severity. It assesses symptoms during the past two weeks on a nine-item checklist with each item scored on a 4-point Likert scale from 0 “Not at all” to 3 “Nearly every day.” From 0 to 27, total scores are read as follows: 0–4 (minimal), 5–9 (mild), 10–14 (moderate), 15–19 (moderately severe), and 20–27 (severe). Consistent with prior research showing 88% sensitivity and 88% specificity for this threshold, this study used a cutoff score of ≥10 that suggests the presence of depressive symptoms [24]. Numerous populations have demonstrated robust psychometric properties on the scale. The Bangla version of the PHQ-9 has demonstrated high concept validity and excellent internal consistency (Cronbach’s α = 0.83) among Bangladeshi university students, thereby establishing its reliability in this context [25]. It has been used conventionally in Bangladesh to assess depression in comparable university populations [26,27]. The PHQ-9 had satisfactory internal consistency in this research, evidenced by a Cronbach’s alpha of 0.816. Finally, we classified the samples into three classes: (i) normal (PHQ-9: 0--4), (ii) depressive symptoms (PHQ-9: 5--9), and (iii) depression (PHQ-9: ≥ 10) [27,28].

### Statistical analysis

Statistical analysis was carried out via SPSS version 25.0. The chi-square test was used to assess the relationships between dependent and independent categorical variables. Finally, a binary multiple logistic regression model was applied to determine the predictors of asthma. Ordinal logistic regression was applied to determine the effect of asthma on depression. The proportional odds assumption (parallel lines) under the model was checked, and the assumption was satisfied. Variance inflation factors (VIFs) were used to check for multicollinearity problems among the independent variables. VIF between 0≤ and ≤5 suggested no multicollinearity problem [29]. The Hosmer and Lemeshow test and Nagelkerke R-square test were used to assess the goodness of fit of the model and its ability to explain the variation in the outcome variable, respectively. We considered a p value <0.05 to indicate statistical significance.

### Ethics approval and consent to participate

The Institute of Biological Science (IBSc), University of Rajshahi, Bangladesh approved this study and provided an ethical clearance letter ((Memo no: 72 (22)/320/ IAMEBBC/ IBSc) to us. We followed all the rules and regulations of the IBSC. Prior to gathering data, we had extensive conversations with our selected participants regarding the aim of our research, and we got informed consent from each individual.

## Results

A total of 400 participants were included in the present study to investigate the prevalence and associated factors of asthma, and the association of asthma with depression among university students in Bangladesh. The average age of the students was 22.6 ± 1.74 years, ranging from 18–28 years. Most of the students were male, unmarried, and the first child of their parents. Almost all the students were non-smokers and did not consume abusive substances. Nearly one-fifth of the students consumed added salt, and a large portion of the respondents consumed red meat fewer than 5 times a month and consumed fried food fewer than 5 times a week. A large number of students did not take part in physical exercise, and almost half of the students exercised 1–2 hours a week. Nearly 30% of the students had a family history of asthma (Table 1).

**Table 1:** Lifestyle, sociodemographic, and behavioral factors of students (n = 400).

Variable	Group	N (%)	Variable	Group	N (%)
**Gender**	Male	250 (62.5)	**Mother’s education**	No schooling	30 (7.5)
				Primary	118 (29.5)
	Female	150 (37.5)		Secondary or above	252 (63)
**Academic year**	1^st^	77 (19.3)	**Father’s education**	No schooling	33 (8.3)
	2^nd^	65 (16.3)		Primary	80 (20)
	3^rd^	114 (28.5)		Secondary or above	287 (71.7)
	4^th^	79 (19.8)	**Residence**	Rural	183 (45.5)
	MS	65 (16.3)		Urban	217 (54.5)
**Monthly family income (BDT)**	Lower (<25K)	201 (50.25)	**Substance abuse**	Yes	27 (6.8)
	Middle (25K-50K)	166 (41.5)		No	373 (93.2)
	Higher (>50K)	33 (8.25)	**Added salt intake**	Yes	68 (17)
**Marital** **status**	Married	34 (8.5)		No	332 (83)
	Unmarried	366 (91.5)	**Red meat intake per month**	No intake	68 (17)
**BMI (kg/m**^**2**^)	Underweight (<18.5 kg/m^2^)	29 (7.2)		1-5 times	283 (70.8)
	Normal (18.5 ≤ BMI < 25)	320 (80)		more than 5 times	49 (12.3)
	Overweight (25 ≤ BMI < 30)	51(12.8)	**Fried Food intake per week**	No intake	19 (4.75)
**Birth order**	First child	187 (46.75)		1-5 times	190 (47.5)
	Second or later child	213 (53.25)		6-10 times	126 (31.5)
				More than 10 times	65 (16.25)
**Father’s occupation**	Farmer	106 (26.5)	**Physical exercise**	No exercise	93(23.3)
	Service	141 (35.3)		1-2 hours	172(43.0)
	Business	119 (29.8)		3 and more hours	135(33.8)
	Others (daily workers, immigrants etc.)	34 (8.4)	**Family** **history of asthma**	Yes	117 (29.3)
				No	283 (70.7)
**Mother’s occupation**	Housewife	342 (85.5)	**Smoking status**	Yes	71 (17.8)
	Non-Housewife	58 (14.5)		No	329 (82.2)

**Note:** BMI: body mass index; K: thousand; BDT: Bangladeshi Taka

### Prevalence of asthma

**Fig 1:**
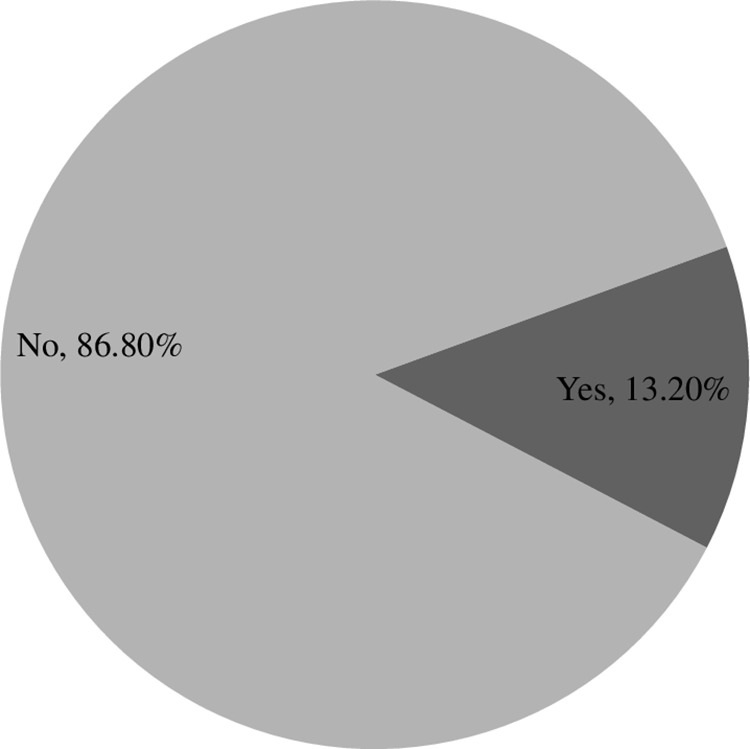
Prevalence of asthma among university students.

The study revealed that the prevalence of asthma among university students of Bangladesh was 53 (13.3%, 95% CI: 10.1% − 17.0%) (Fig 1). Asthma was shown to be more prevalent among students in urban areas than in students from rural areas. Underweight students were more likely to develop asthma than normal and overweight students. Students who were born as second- or later-born children had a greater prevalence of asthma than students born as the first child. The prevalence of asthma was found to be greater among students from higher-income families than among their counterparts. Asthma was found to be more common among smokers than among nonsmokers. Students who consumed fried food more than ten times a week and red meat more than five times a month were found to have a higher prevalence of asthma than their counterparts. It was shown that students with a family history of asthma had a greater prevalence of the disease than students who did not have asthma. The chi-square test revealed that the associations between the above variables and asthma were statistically significant. The remaining variables were not significantly associated with asthma (Table 2).

**Table 2:** Association between lifestyle, socio-demographics, and behavioral factors and asthma among university students.

Variable	Group	Are you suffering from asthma (diagnosed by a doctor within one year)?		Chi-square value	p-value*
		No	Yes		
**Gender**	Male	217 (86.8%)	33 (13.2%)	0.026	0.980
	Female	130 (86.7%)	20 (13.3%)		
**Marital status**	Unmarried	318 (86.9%)	48 (13.1%)	0.069	0.791
	Married	29 (85.3%)	5 (14.7%)		
**Residence**	Urban	180 (82.9%)	37 (17.1%)	5.961	0.017
	Rural	167 (91.3%)	16 (8.7%)		
**BMI (kg/m**^**2**^)	Underweight (<18.5 kg/m^2^)	21 (72.4%)	8 (27.6%)	6.536	0.038
	Normal (18.5–24.9 kg/m^2^)	279 (87.2%)	41 (12.8%)		
	Overweight (≥25 kg/m^2^))	47 (92.2%)	4 (7.8%)		
**Birth order**	First Child	170 (90.9%)	17 (9.1%)	5.285	0.026
	Second or later child	177 (83.1%)	36 (16.9%)		
**Father’s occupation**	Farmer	95 (89.6%)	11 (10.4%)	6.166	0.104
	Service	122 (86.5%)	19 (13.5%)		
	Business	105 (88.2%)	14 (11.8%)		
	Others (daily worker)	25 (73.5%)	9 (26.5%)		
**Mother’s occupation**	Housewife	297 (86.8%)	45 (13.2%)	0.017	0.836
	Non-Housewife	50 (86.2%)	8 (13.8%)		
**Father’s education**	Illiterate	29 (87.9%)	4 (12.1%)	0.104	0.949
	Primary	70 (87.5%)	10 (12.5%)		
	Secondary and above	248 (86.4%)	39 (13.6%)		
**Mother’s education**	Illiterate	27 (90%)	3 (10%)	2.077	0.354
	Primary	98 (83.1%)	20 (16.9%)		
	Secondary and above	222 (88.1%)	30 (11.9%)		
**Monthly family income (BDT)**	Lower class (<25K)	170 (84.6%)	31 (15.6%)	10.711	0.005
	Middle class (25K-50K)	153 (92.2%)	13 (7.8%)		
	Higher class (>50K)	24 (72.7%)	9 (27.3%)		
**Smoking status**	No	293 (89.1%)	36 (10.9%)	8.588	0.003
	Yes	54 (76.1%)	17 (23.9%)		
**Substance abuse**	No	324 (86.9%)	49 (13.1%)	0.062	0.770
	Yes	23 (85.2%)	4 (14.8%)		
**Added salt intake**	No	291 (87.7%)	41 (12.3%)	1.378	0.242
	Yes	56 (82.4%)	12 (17.6%)		
**Red meat intake per month**	No intake	59 (89.4%)	7 (10.6%)	9.406	0.009
	1-5 times	245 (88.8%)	31 (11.2%)		
	more than 5 times	43 (74.1%)	15 (25.9%)		
**Fried food intake per week**	No intake	15 (78.9%)	4 (21.9%)	8.451	0.038
	1-5 times	168 (88.4%)	22 (11.6%)		
	6-10 times	114 (90.5%)	12 (9.5%)		
	more than 10 times	50 (76.9%)	15 (23.1%)		
**Physical exercise**	No exercise	80(86.0)	13(14.0)	2.479	0.290
	1-2 hours	145(84.3)	27(15.7)		
	3 and more hours	122(90.4)	13(9.6)		
**Family history of asthma**	No	275 (97.2%)	8 (2.8%)	91.448	0.000
	Yes	72 (61.5%)	45 (38.5%)		

**Note:** *p value estimated using chi-square test

### Predictors of asthma

Only significantly associated factors determined by the chi-square test were considered independent variables in the binary multiple logistic regression model. The VIF showed that there was no evidence (0 ≤ VIF ≤ 5) of multicollinearity problems among the independent variables. After adjusting for other variables, the model demonstrated that underweight students were 17.26 times more likely to have asthma than overweight students (p < 0.01). Compared to students who were born in the first order, those who were born in the second order or later were more likely to have asthma (3.364 times higher) (p < 0.01). Compared to students who did not have a family history of asthma, those with a family history of asthma were 36.514-fold more likely to have asthma (p < 0.01). The Hosmer and Lemeshow test showed that the proposed model was well-fitted, and the Nagelkerke R square value indicated that the model could explain 52.4% of the variation in the outcome variable (Table 3).

**Table 3:** Predictors of asthma among university students (binary logistic regression model).

Variable and category	B	p value	aOR (95% CI)	VIF
**Residence, Urban Vs Rural** ^**R**^	0.638	0.129	1.892 (0.831-4.309)	1.049
**BMI Category**		0.004		1.008
**Underweight Vs Overweight** ^**R**^	2.848	0.002	17.260(2.772-20.490)	
**Normal weight Vs Overweight** ^**R**^	0.977	0.183	2.656(0.631-11.184)	
**Order of birth,** **2**^**nd**^ **or later child Vs 1**^**st**^ **child**^**R**^	1.213	0.004	3.364(1.472-7.688)	1.026
**Monthly family income (BDT)**		0.175		1.014
**Middle Vs Lower** ^**R**^	−0.809	0.064	0.445(0.189-1.049)	
**Higher Vs Lower** ^**R**^	−0.102	0.886	0.903(0.223-3.661)	
**Smoking status, Yes Vs No** ^**R**^	0.729	0.139	2.074(0.788-1.039)	1.039
**Red meat intake per month**		0.160		1.118
**1-5 times Vs No intake** ^**R**^	−0.325	0.569	0.723(0.236-2.211)	
**More than 5 times Vs No intake** ^**R**^	0.684	0.326	1.982(0.506-7.758)	
**Fried food intake per week**		0.705		1.092
**1-5 times Vs No intake** ^**R**^	−0.882	0.346	0.414(0.066-2.595)	
**6-10 times Vs No intake** ^**R**^	−1.105	0.246	0.331(0.051-2.139)	
**more than 10 times Vs No intake** ^**R**^	−0.795	0.416	0.452(0.067-3.063)	
**Family history of asthma, Yes Vs No** ^**R**^	3.598	0.001	36.514(13.881-96.051)	1.021
**Hosmer and Lemeshow Test**	Chi-square test-value = 9.040, p value = 0.339			
**Nagelkerke R Square-value** = 0.50				

**Note:** B: Regression coefficients, R: reference category, CI: confidence interval, aOR: adjusted odds ratio, VIF: variance inflation factor

### Association of asthma with depression symptoms

**Fig 2:**
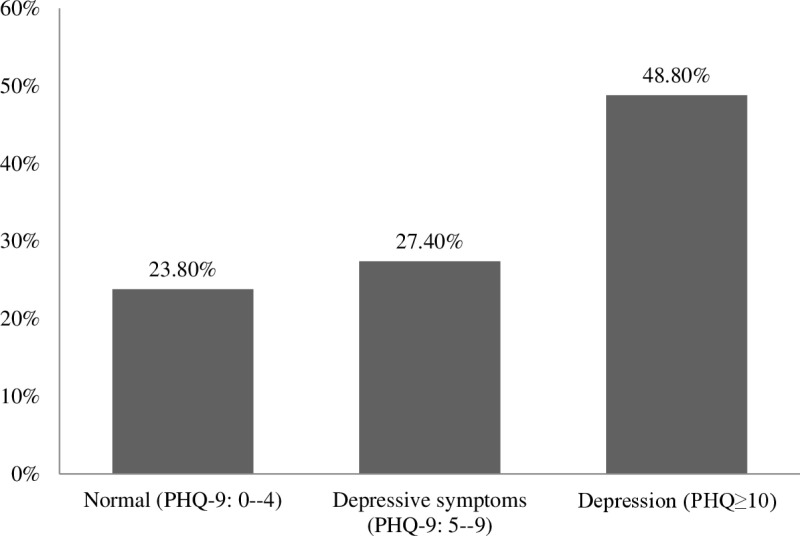
Prevalence of depression in university students.

**Fig 3:**
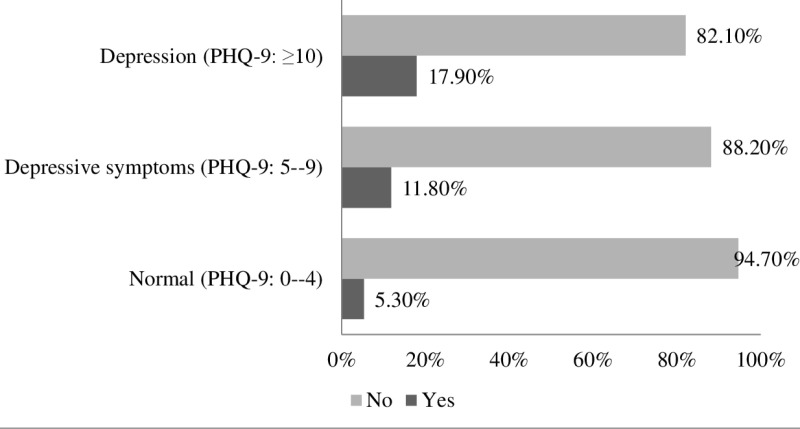
The prevalence of asthma among students with different types of depression.

The results revealed that 195(48.8%) students were suffering from depression while 110(27.4%) students had depressive symptoms (Fig 2). A higher prevalence of asthma was observed among students with depression (17.90%) compared to those without depression (5.30%), indicating a potential association between depressive symptoms and asthma (Fig 3). A chi-square test revealed that the association between asthma and depression in students was significant (p < 0.01). After the effects of socioeconomic, demographic and anthropometric factors were adjusted, the ordinal logistic regression analysis demonstrated that students with asthma had a 2.656-fold greater chance of having depression compared to students who did not have asthma (Table 4).

**Table 4:** Ordinal logistic regression analysis of the influence of asthma on depression status among university students.

Independent variable		B	SE	p value	aOR (95% CI)
**Asthma**	Yes	0.977	0.320	0.002	2.656(1.418-4.978)
	No^R^	0			1

**Note:** B, regression coefficient; SE, standard error; aOR, adjusted odds ratio; CI, confidence interval. Adjusted gender, residence, order of birth, family monthly income, body mass index, parents’ occupation, parents’ education

## Discussion

### Prevalence of asthma

In this study, we sought to investigate the prevalence of asthma and its associated factors. We found that 13.30% of university students in Bangladesh were suffering from asthma, which was higher (10.7%) than that reported in a previous study in Bangladesh; this study was conducted with undergraduate students at Bangladesh Agricultural University, Bangladesh, from September 2013 to October 2013 [12]. The prevalence of asthma in middle- and low-income countries is increasing at a concerning rate [30] which calls for further large-scale investigations. A previous study reported that the prevalence of asthma among rural Bangladeshi communities was 9.69%, and the authors recruited participants aged 15─60 years [9]. Another Bangladeshi study involving rural people in Tangail district in Bangladesh was conducted from January to June 2019, and the prevalence of asthma was reported to be 4.2% [8]. The asthma prevalence in the present study was greater than that in university students in other countries, such as Turkey (2.3%) [31], Thailand (8.8%) [32], Poland (9.6%) [33], and Kuwait (11.9%) [34]. The most important causes of asthma are health care, air pollution and economic stability [35]. The limited health care facilities, greater air pollution and lower economic stability in Bangladesh compared to other four countries are possible reasons for the higher prevalence of asthma among universities in Bangladesh.

### Predictors of asthma

We found that a family history of asthma was the most important predictor of asthma. The same results were reported in other studies with university students [36–38]. This finding was also congruent with some Bangladeshi studies with different populations [8,10,39]. The Centers for Disease Control and Prevention (CDC) reported that three-fifths of asthma cases are hereditary [40]. The present study revealed that underweight students were more likely to have asthma than overweight students, and a study with medical students reported similar findings [41]. However, these results contradict those of other studies [42–44], which revealed that overweight university students were more likely to have asthma than underweight university students.

In the present study, we observed that the order of birth was another influencing factor of asthma. Students who were born second or later were more prone to develop asthma than their counterparts, and the same finding was also reported in the literature [45]. In this study, a logistic regression model demonstrated that smoking status was not a significant predictor of asthma; however, the chi-square test revealed that a greater number of smokers had asthma compared to nonsmokers and the association between smoking status and asthma was significant. Some previous studies reported that smoking status was not a predictor of asthma, although several epidemiological studies have shown that smokers are more likely to have asthma than nonsmokers [34,37,38].

### Association of asthma with depression symptoms

This study found that approximately half of students have symptoms of depression (48.80%). Globally, it was lower than other countries; Gambia 68.1% [46]; Kenya 57.7% [47]; Pakistan 56% [48]; and higher than Malaysia (45%) [49]; Egypt 42% [50]. A similar previous study reported that the prevalence of depression among Bangladeshi university students was 47.3% and higher in female than male students (50.7% vs. 43.6%) that was conducted from April to September 2018 [26]. Another study among students of Jahangirnagar University in Bangladesh was conducted from December 8, 2019 to January 23, 2020 found that female students have more levels of depression than their counterparts (86.1% vs 82.2%) [51]. These results emphasize the importance of gender-sensitive mental health interventions in university settings.

The study also found a significant association between asthma and depression. Depression is the most prevalent comorbidity among asthmatic patients, and it has a significant effect on treatment, and research indicates that asthma increases the risk of depression [52]. The study examined the association of asthma and depression and revealed that 17.9% of asthma participants had depressive symptoms compared to those without depression (5.30%), and this relationship was statistically significant (p < 0.01), which is similar to that reported by Stubbs and colleagues, who reported that 27.5% of participants had difficult asthma [48,53,54], and another study by Oga et el found that 22.3% of asthmatic patients have depression [55]. Another similar previous study reported that 18.1% of adults with asthma had comorbid depressive disorders [15]. In the Bangladeshi context, a significant correlation was found between depression and asthma among university students, and a similar relationship was observed in a previous study among the 18–82-year-old population [56]. Future studies ought to investigate the associations between asthma and depression as well as the efficacy of combined treatments for managing both diseases. The combined approach of physical and mental health treatment in Bangladesh could be a key tool to improve the general health and quality of life for university students.

### Strengths and limitations of the study

Among Bangladeshi university students, asthma is a significant but understudied condition. This study is one of the first to investigate this phenomenon. It is a valuable resource for understanding student’s health issues, as it employs appropriate statistical methods to analyze asthma related characteristics. The reliability and comprehensiveness of the results are enhanced by precise analytical procedures. The study, however, has limitations. It is a cross-sectional study, cannot establish a causal relationship between asthma and other factors. Furthermore, using self-reported data subject to recall bias.

## Conclusion

The current study examined the prevalence of asthma and its contributing factors among university students in Bangladesh. The findings indicate that a significant number of these students are affected by asthma, with certain modifiable factors linked to the condition. It was also found that about half of the students showed symptoms of depression. Additionally, depression is a common comorbidity among Bangladeshi university students with asthma, highlighting the need for further research to explore the underlying mechanisms and to create effective management strategies for both asthma and depression. These insights will aid in the development and implementation of campus-based treatment programs, peer education initiatives, and a national surveillance system aimed at controlling asthma. Moreover, it is essential for university authorities to effectively establish and reinforce the concerned government regulations.

## Supporting information

S1 Data Data. (SAV)

## Abbreviations

BMI: body mass index
CI: confidence interval
SPSS: Statistical Package for Social Sciences
WHO: World Health Organization
B: regression coefficients
aOR: adjusted odds ratio
R: reference category
VIF: variance inflation factor
LMICs: low- and middle-income countries
PEN: Package of Essential Noncommunicable Disease Interventions
K: Thousand
BDT: Bangladeshi Taka
CDC: Centers for Disease Control and Prevention
